# Glymphatic System in the Central Nervous System, a Novel Therapeutic Direction Against Brain Edema After Stroke

**DOI:** 10.3389/fnagi.2021.698036

**Published:** 2021-08-04

**Authors:** Xiangyue Zhou, Youwei Li, Cameron Lenahan, Yibo Ou, Minghuan Wang, Yue He

**Affiliations:** ^1^Department of Neurosurgery, Tongji Hospital, Tongji Medical College, Huazhong University of Science and Technology, Wuhan, China; ^2^Burrell College of Osteopathic Medicine, Las Cruces, NM, United States; ^3^Department of Neurology, Tongji Hospital, Tongji Medical College, Huazhong University of Science and Technology, Wuhan, China

**Keywords:** cerebral spinal fluid, glymphatic system, brain edema, stroke, perivascular space, aquaporin 4, inflammatory cytokine

## Abstract

Stroke is the destruction of brain function and structure, and is caused by either cerebrovascular obstruction or rupture. It is a disease associated with high mortality and disability worldwide. Brain edema after stroke is an important factor affecting neurologic function recovery. The glymphatic system is a recently discovered cerebrospinal fluid (CSF) transport system. Through the perivascular space and aquaporin 4 (AQP4) on astrocytes, it promotes the exchange of CSF and interstitial fluid (ISF), clears brain metabolic waste, and maintains the stability of the internal environment within the brain. Excessive accumulation of fluid in the brain tissue causes cerebral edema, but the glymphatic system plays an important role in the process of both intake and removal of fluid within the brain. The changes in the glymphatic system after stroke may be an important contributor to brain edema. Understanding and targeting the molecular mechanisms and the role of the glymphatic system in the formation and regression of brain edema after stroke could promote the exclusion of fluids in the brain tissue and promote the recovery of neurological function in stroke patients. In this review, we will discuss the physiology of the glymphatic system, as well as the related mechanisms and therapeutic targets involved in the formation of brain edema after stroke, which could provide a new direction for research against brain edema after stroke.

## Introduction

Brain edema refers to the pathological phenomenon in which the water and volume in the brain increase. After the central nervous system (CNS) is damaged, such as in stroke, trauma, intracranial space-occupying lesions, inflammatory reactions, and metabolic disorders, the blood-brain barrier (BBB) is destroyed, and water and macromolecular substances exudate and accumulate in the perivascular and interstitial cells. Brain tissue hypoxia, cell membrane dysfunction, or intracellular electrolyte and osmotic changes lead to intracellular swelling in the damaged brain. In the damaged nervous system, cerebrospinal fluid (CSF) circulation pathway obstruction causes ventricular enlargement or periventricular leukoencephalopathy. Brain edema after stroke increases intracranial pressure (ICP), which in turn aggravates brain edema, causes functional and structural damage to brain tissue, leads to the occurrence of epilepsy, paralysis, aphasia, and other brain injury symptoms. With the further aggravation of brain edema or diffuse progression, brain herniation and brain stem damage occur, eventually leading to brain death. Therefore, timely and effective control of brain edema is beneficial to improve the symptoms and prognosis of stroke patients. Unlike other components of the body, the brain was believed to lack lymphatic vessels. However, recent studies have found that the glymphatic system transports metabolic waste and regulates CSF flow (Rennels et al., 1985, 1990; Iliff et al., 2012). The formation of brain edema is closely related to CSF circulation. Therefore, the glymphatic system plays an important role in the formation and recovery of brain edema through its transport pathway. This means that an understanding of the mechanism of the glymphatic system in brain edema after stroke can provide a new target for the treatment, thus promoting the recovery of neurological function and improving the prognosis of patients after stroke. Next, we will discuss the role of the glymphatic system in the pathophysiological mechanism of the formation and regression of brain edema after stroke. We will also provide a brief introduction to the current therapeutic drugs that regulate the glymphatic system in the treatment of brain edema.

## Physiology of The Glymphatic System in The CNS

### The Glymphatic System in CNS

The lymphatic system plays an important role in fluid homeostasis, lipid metabolism, and immune control for health, but the brain was thought to be the only organ that lacks lymphatic vessels (Trevaskis et al., 2015). However, the brain has an extremely high density of cells, with high metabolic activity that is disproportionate to its size, and which produces a large number of metabolites (Nedergaard, 2013). This introduces questions regarding the mechanisms by which the brain clears fluid and waste products, as well as by how it facilitates the influx of immune cells into the brain. The brain may have a different metabolic-clearing pathway than other tissues. The discovery of the perivascular space and the glymphatic system provides a solution for this contradiction (Iliff et al., 2012). Blue dextran 2000 injected into the caudate nucleus of rats (Cserr and Ostrach, 1974) and the distribution of horseradish peroxidase (HRP; Cserr et al., 1977) can be observed along the perivascular space, and may flow into the CSF. Similar results were obtained through an experiment conducted in the different regions of the brain, such as the internal capsule, midbrain, and inferior colliculus (Szentistványi et al., 1984; Ball et al., 2010). To summarize, all of these studies suggest that the CSF in the subarachnoid space is facilitated by arterial pulsation (Mestre et al., 2018b) into the brain along the perivascular space surrounding the cerebral artery. It enters the brain interstitium through aquaporin 4 (AQP4) on the perivascular astrocytic endfeet (Mestre et al., 2018a), and then interstitial fluid (ISF), which contains metabolic waste, enters the paravenous vascular space (Abbott, 2004). Thus, the perivascular space in the brain parenchyma and the aquaporin on astrocytes together constitute the glymphatic system.

AQP4 is an important bridge in the glymphatic system (Iliff and Simon, 2019). CSF and ISF are mainly exchanged through AQP4, but the specific exchange mechanism between the two fluids still requires further study. The perivascular spaces are important channels for CSF entering into and flowing out of the brain parenchyma, and metabolic wastes in the brain are also eliminated through it (Wardlaw et al., 2020). In the physiological condition, the glymphatic system facilitates the clearance of metabolic wastes from the brain and helps maintain homeostasis of the CNS, which depends on the anatomic integrity of the perivascular network (Iliff et al., 2012). Interestingly, when damage to the CNS occurs, such as hemorrhagic (Luo et al., 2016; Goulay et al., 2017; Golanov et al., 2018) or ischemic stroke (Gaberel et al., 2014; Wang et al., 2017), the flow in the glymphatic system is often significantly decreased for a period of time, and the degree and speed of recovery of the glymphatic system correlates with the prognosis of stroke (Goulay et al., 2017; Wang et al., 2017). The above mentioned suggests that the glymphatic system may have an important role in the progression and recovery of stroke.

### The Clearance Pathway of the Glymphatic System

**Figure 1:**
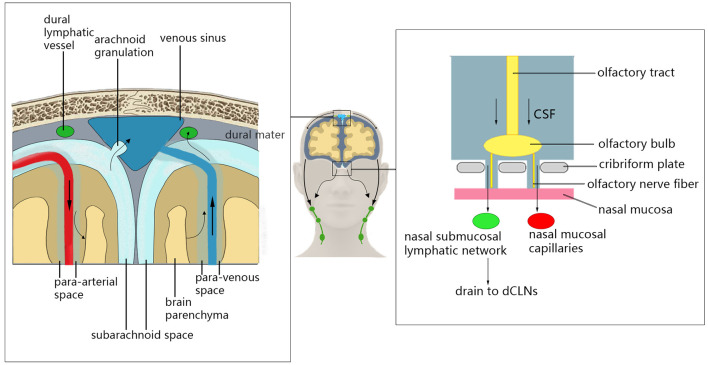
Glymphatic system clearance pathway. Cerebrospinal fluid (CSF) can enter the dural venous sinus through the unidirectional flow of arachnoid particles, and then leave the central nervous system (CNS). The CSF can reach the nasal mucosa along the olfactory nerve through the ethmoid plate and can accumulate in the deep cervical lymph node through the lymphatic network under the nasal mucosa. Additionally, the CSF may enter the blood through the microvascular structure in the nasal mucosal. CSF can also enter deep cervical lymph nodes through the lymphatic vessels near the dural sinus.

In addition to transporting metabolic waste from the brain parenchyma through the glymphatic system, there are other clearance pathways in the brain that remove metabolic waste from the CNS (Louveau et al., 2017; Mestre et al., 2020b; Figure 1). CSF and some ISF can enter the dural venous sinus through the unidirectional flow of arachnoid particles, and can then leave the CNS (McLone, 1980). This process may provide nutrients for axons/neurons while removing harmful metabolites (Killer, 2013). Recent studies have shown that CSF and solute flow in the surrounding space of the vagus and olfactory nerve (Iliff et al., 2013a) through the olfactory tract–ethmoid plate–nasal submucosa pathway (Bradbury and Cole, 1980). The CSF of mice and humans can reach the nasal mucosa along the olfactory nerve through the ethmoid plate and can accumulate in the deep cervical lymph node through the lymphatic network under the nasal mucosa. Additionally, the CSF may enter the blood through the microvascular structure in the nasal mucosa (Sakka et al., 2011). This pathway may play an important role in regulating intracranial volume, metabolic waste efflux, and neuroimmune function in the CNS (Jones, 2004; Tenenbaum et al., 2013). Recently, studies conducting immunohistochemical staining of mouse dural tissue have revealed the presence of lymphatic vessels near the dural sinus of mouse brain tissue, which was thought to lack lymphoid tissue (Aspelund et al., 2015; Louveau et al., 2015b). These lymphatic vessels near the dural sinus are associated with blood vessels and cranial nerves and are connected to the extracranial lymphatic vessels through the corresponding pores of the skull base. In the mouse animal model, a fluorescent tracer was injected into the brain parenchyma to find that the tracer flowed to the paranasal meningeal lymphatic vessels (Ahn et al., 2019), and then into the deep cervical lymph nodes. Conversely, no tracer was observed when ligating the lymphatic vessels flowing to the deep cervical lymph nodes, and the diameter of the meningeal lymphatic vessels was increased (Louveau et al., 2015b). In mice with knockout dural lymphatic vessels, there was significantly reduced draining of the tracer into the deep cervical lymph nodes (Aspelund et al., 2015). Visualized studies on the glymphatic system and meningeal lymphatic vessels by imaging methods have verified that the meningeal lymphatic vessels are downstream of the glymphatic pathway in humans (Zhou et al., 2020). Therefore, the dural lymphatic vessels are important pathways for the removal of intracranial solutes and CSF.

### The Influencing Factors of the Glymphatic System

The glymphatic system is regulated by various factors. An important factor is the arterial pulsation that is related to the heart cycle. It was found that the influx of CSF into the brain parenchyma and the outflow of ISF from brain parenchyma were significantly reduced by unilateral ligation of the internal carotid artery (ICA) of mice, and it eliminated the arterial pulsatility. Conversely, the CSF influx was significantly increased after the pulsatility of penetrating arteries was enhanced, resulting from systemic administration of the adrenergic agonist, dobutamine (Iliff et al., 2013b). In another study, the influx of CSF was influenced by the arterial pulsation that was related to respiration (Dreha-Kulaczewski et al., 2015). They applied a novel high spatial and temporal resolution real-time magnetic resonance imaging (MRI) technique and observed high CSF flow being elicited during every inspiration in healthy people. However, only a minor flow component could be attributed to cardiac pulsation (Dreha-Kulaczewski et al., 2015). The generation of CSF is also an important driving force for CSF flow in the glymphatic system. A significant decrease in CSF influx with a decrease of solute outflow in ISF was observed after acetazolamide administration, which can reduce the production of CSF by inhibiting the carbonic anhydrase in the choroid plexus (Lundgaard et al., 2017). The level of consciousness and the body position also affect the inflow of CSF in the glymphatic system (Benveniste et al., 2019). The study found that sleep or anesthesia in mice was associated with increased fluid flow in the peripheral CSF tracer and improved solute clearance between tissues (Xie et al., 2013). Fluorescent CSF tracers that flow into the brain while in the lateral decubitus position were higher than in the supine position (Lee et al., 2015). Using Dexmedetomidine can enhance the slow wave activity of EEG and enhance the convection of CSF and ISF in the glymphatic system to ensure that the intrathecal drug can reach the brain parenchyma more effectively (Lilius et al., 2019). Age is also an influencing factor of the glymphatic system. In an observational cohort study, it was found that the glymphatic system was damaged during the aging process (Zhou et al., 2020).

## The Molecular Mechanisms of Brain Edema Formation After Stroke

Brain edema is characterized as an excessive accumulation of fluid in the brain tissue. It can result from a variety of CNS injuries, such as brain trauma, hemorrhagic and ischemic stroke, etc. (Walcott et al., 2012). Current studies often classify it as cytotoxic, ionic, or vasogenic edema (Simard et al., 2007). Energy consumption resulting from dysfunction will consequently lead to cell swelling, namely cytotoxic edema (Thrane et al., 2011). Ionic edema is a massive redistribution of water and solute between tissues and interstitial spaces through a variety of water and ion channels (Rungta et al., 2015). Vasogenic edema has been attributed to the infiltration of proteins and ions from plasma into cells following the destruction of the BBB (Nag et al., 2009), which can lead to the expansion of the interendothelial cell space and further aggravation of edema (Daneman and Prat, 2015). In many diseases, the type of edema periodically changes over time. Moreover, due to the focal nature of the site of edema, heterogeneity in the type and extent of edema may occur in different regions of the same brain tissue simultaneously.

### Brain Edema After Ischemic Stroke

In the early stage of ischemic stroke, the BBB remains relatively intact, and only cytotoxic edema occurs due to insufficient perfusion of blood flow and depletion of adenosine triphosphate (ATP) in cells (Thrane et al., 2011). Three hours after ischemic stroke, the tight junction (TJ) of the BBB is destroyed (Belayev et al., 1996; Ren et al., 2013), allowing water, ions (Daneman and Prat, 2015), and albumin in plasma to permeate into the perivascular space and brain parenchyma along the hydrostatic pressure gradient, resulting in further swelling of brain tissue. However, the experimental results show that there is no temporal correlation between the direct osmotic effect of albumin exudation and the formation of edema (Menzies et al., 1993), which suggests that there may be other mechanisms in vasogenic edema that lead to brain swelling. Inflammation is a powerful mechanism that triggers and enhances brain edema, including further injury of BBB, induction of leukocyte migration, production of cell debris (Ivens et al., 2007; Ralay Ranaivo et al., 2012), and the accumulation of perivascular immune complexes (Carare et al., 2013). When the BBB is intact, most of the immunoreactive cells do not enter the brain parenchyma (Daneman and Prat, 2015). However, in addition to neurons, cells with major immune and supportive functions, such as endothelial cells, microglia, astrocytes, and pericytes, gather in the perivascular space (Williams et al., 2001; Rangroo Thrane et al., 2013; Faraco et al., 2017). Microglia can be activated by factors released *via* activated monocytes, lymphocytes, and other activated microglia. Meanwhile, the activated microglial cells also release cytokines, such as vascular endothelial growth factor-A (VEGF-A), interleukin-1 (IL-1), tumor necrosis factor-α (TNF-α; Chen et al., 2019), transforming growth factor β (TGFβ), matrix metalloproteinase9 (MMP9), and MMP14 (Holmin and Mathiesen, 2000; Witt et al., 2008; Argaw et al., 2012; Vinnakota et al., 2013), which promote the aggregation and activation of peripheral immune cells. The inflammatory cytokines can lead to expansion of the inflammatory response cascade, which may lead to increased permeability of BBB and aggravation of brain edema, and is closely related to the poor prognosis of stroke (Koerner et al., 2007; Sharma et al., 2013).

### Brain Edema After Hemorrhagic Stroke

After a hemorrhagic stroke, the formation and stimulation of blood clots facilitate the reduction of hydrostatic pressure around the hematoma and the exudation of plasma proteins to cause vasogenic brain edema (Zheng et al., 2016). After blood flows into the brain tissue, it activates the coagulation cascade through thrombin, which is not only cytotoxic but can directly cause cell death (Donovan et al., 1997) and can activate the thrombin receptor to make endothelial cells contract and allow TJs to open (Guan et al., 2004), thereby increasing the permeability of the BBB and aggravating vasogenic edema. In addition, red blood cell lysate is also an important contributor to brain edema after a hemorrhagic stroke. The main products of red blood cell lysis are hemoglobin, heme, and iron. Both hemoglobin (Wang Y.-C. et al., 2014) and heme (Lin et al., 2012) can induce inflammation after bleeding. Iron overload can cause neurotoxicity (Hua et al., 2007), and injection of deferoxamine can reduce hydrocephalus caused by red blood cell lysate (Gao et al., 2014). When the BBB is severely damaged or opens in the early stage of hemorrhagic stroke, the immune cells in the blood vessels, such as T cells (Kunis et al., 2013) and neutrophils (Wang et al., 2016), may also enter the perivascular space of the brain parenchyma, and can be induced by antigen-presenting cells, such as activated microglia and astrocytes (Xue and Yong, 2020), to proliferate, differentiate, and release proinflammatory cytokines, which will exacerbate the destruction of the BBB and the formation of vasogenic edema.

## The Role of The Glymphatic System in The Formation of Brain Edema After Stroke

**Figure 2:**
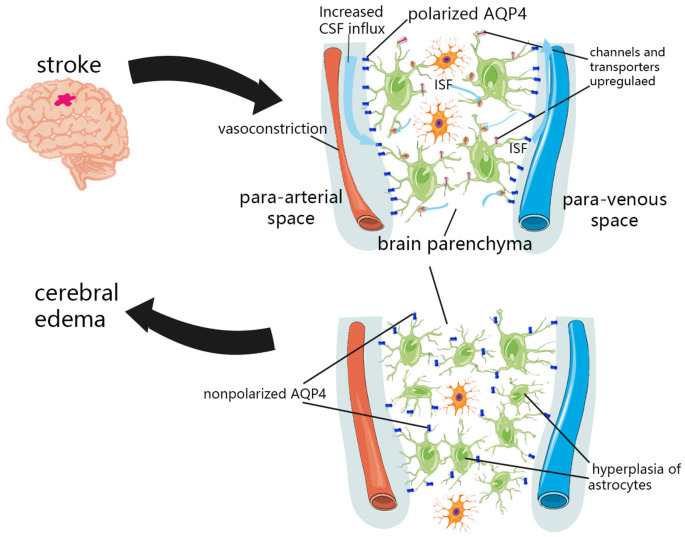
Glymphatic system dysfunction in brain edema after stroke. In the initial stage of ischemic stroke, the decrease in tissue blood supply can cause spreading depolarization of cortical neurons and astrocytes, thus triggering vasoconstriction and abnormal internal flow of CSF. The expression of sodium transporter NKCC1 Sulfonylurea receptor 1 (SUR1)-Regulated Nonselective Cation Channels (NCCa-ATP) were upregulated on the cell surface, which promotes the transport of ions and water to the brain parenchyma. Due to the hyperplasia of astrocytes and the loss of AQP4 polarization, the influx and outflow in the glymphatic system decreased, and the clearance of solutes and water decreased after a period of CNS injury.

Recently, the theory of the perivascular space and associated glymphatic systems may challenge our theoretical hypothesis of the formation of brain edema, suggesting that the external fluid of brain edema originates from the perivascular CSF (Iliff et al., 2012; Figure 2). Within minutes of ischemic stroke, brain tissue begins to swell, and water content is increased (van der Toorn et al., 1996; Mestre et al., 2020a). Concurrently, CSF flows rapidly into brain tissue along the perivascular space of mice (Iliff et al., 2012). This suggests that the external perfusion required for brain tissue swelling, at least partially, comes from CSF rather than intravascular fluid. This theory also explains the contradiction of the inconsistency between the timing of when BBB integrity is destroyed and when brain edema occurs. Moreover, the BBB usually recovers earlier than the resolution of brain edema (Wang et al., 2017). An important part of the glymphatic system, the AQP4 channel, has been shown to change the location from the endfeet of perivascular astrocytes to other places after CNS injury (Iliff et al., 2014; Kress et al., 2014; Peng et al., 2016; Wang et al., 2017), and the recovery of this localization change coincides with the recovery time of brain edema (Iliff et al., 2014). This indicates that the glymphatic pathway may be an important component of fluid outflow in the recovery stage of brain edema. The glymphatic system is also essential in vasogenic edema. Astrocytes, microglia, pericytes, endothelial cells, and leukocytes can interact directly in the perivascular space and brain parenchyma along the glymphatic pathway (Kivisäkk et al., 2003; Arumugam et al., 2009), and inflammatory cytokines can also spread through this pathway (Chodobski et al., 2003; Dimitrijevic et al., 2007; Scholz et al., 2007; Hatterer et al., 2008). Although inflammation and edema may be detrimental in the short–term due to space constraints, this process is also necessary for removing cell debris, and may help repair and close the BBB more quickly (Willis et al., 2013). The role of the glymphatic system in the formation and elimination of brain edema may be multidimensional, and its complexity remains under further investigation.

### Pathophysiological and Molecular Mechanisms of the Glymphatic System in Ischemic Stroke

#### Increased CSF Influx

The conventional viewpoint is that the perfusion needed for ionic edema comes from vessels (Papadopoulos and Verkman, 2013). However, the discovery of the perivascular space and glymphatic pathways challenges this view (Iliff et al., 2012), and studies have shown that within minutes of ischemic stroke, CSF flows rapidly into brain tissue along the perivascular space (Mestre et al., 2020a). This highlights the important role of the glymphatic system in cerebral edema in ischemic stroke.

The redistribution of water and osmotic ions, such as sodium, caused by cytotoxic edema is the driving force for further ionic edema, resulting in swelling of brain tissue (Simard et al., 2007). In the initial stage of ischemic stroke, the decrease in tissue blood supply can cause spreading depolarization of cortical neurons and astrocytes, thus triggering vasoconstriction and abnormal internal flow of CSF (Mestre et al., 2020a). This abnormal internal flow may also be related to a variety of factors. It was found in pathological tissues that insufficient blood perfusion could induce capillary contraction and microvascular collapse of pericytes (Hall et al., 2014), which may dilate the perivascular space and reduce the resistance of CSF inflow. More CSF flows into the perivascular space, which then flows into the brain tissue through glymphatic pathways. Various ion channels and transporters located in the plasma membrane of astrocytes also facilitate the influx of permeable ions and water. After the CNS was damaged, the expression of sodium transporter NKCC1, Sulfonylurea receptor 1 (SUR1)-Regulated Nonselective Cation Channels (NCCa-ATP) were upregulated on the cell surface (Mies et al., 1991; Simard et al., 2006), which promoted the transport of ions and water to brain parenchyma. In addition, the regulation of glymphatic pathway flow is affected by cerebral artery pulsation (Iliff et al., 2013b). Enhanced cerebral artery pulsation in patients with acute ischemic stroke may also increase the influx of CSF to the brain parenchyma, and may aggravate cerebral edema in the early stage of stroke (He et al., 2014).

#### Hyperplasia of Astrocytes and Loss of AQP4 Polarization

Although studies have confirmed that in the initial stage of ischemic stroke, an increase in the influx flow occurs, which facilitates the flow of ions and water from the subarachnoid CSF into the brain parenchyma (Mestre et al., 2020a). However, several experiments in humans and mice have shown that the influx and outflow in the glymphatic system decreased and that the clearance of solutes and water decreased after a period of CNS injury (Gaberel et al., 2014; Goulay et al., 2017; Wang et al., 2017; Golanov et al., 2018). This is most likely associated with glial hyperplasia after CNS injury and the loss of AQP4 polarization (Iliff et al., 2014; Kress et al., 2014; Peng et al., 2016; Wang et al., 2017). AQP4 and astrocytes are important components of the glymphatic system.

Extensive astrocytic hyperplasia can be observed in the lesion area in a local brain microinfarction mouse model (Wang et al., 2017). Concurrently, two-photon microscope *in vivo* imaging showed that the tracer inflow in the ipsilateral glymphatic system decreased (Wang et al., 2017). In another study, decompressive craniectomy failed to improve the decreased glymphatic influx flow caused by ischemic infarction (Iliff et al., 2014). This suggests that astrocytic hyperplasia, accompanied by the loss of AQP4 polarization, may impair the influx flow of the glymphatic system, which is independent of fluid hydrostatic pressure.

AQP4 is the most widely distributed aquaporin in the CNS (Nagelhus and Ottersen, 2013; Welberg, 2013). AQP4 is mainly distributed in astrocytes, ependymal epithelial cells, and choroid plexus epithelial cells. Additionally, it is abundantly expressed in the arachnoid, pia mater, capillary, and other adjacent astrocytic endfeet (Nagelhus and Ottersen, 2013). After CNS damage, such as cerebral infarction (Gaberel et al., 2014; Wang et al., 2017), hemorrhage (Goulay et al., 2017; Golanov et al., 2018), or trauma (Iliff et al., 2014), the expression and distribution of AQP4 is often changed and is accompanied by decreased CSF influx. In the physiological state, the polar distribution of AQP4 in astrocytic endfeet is believed to facilitate the flow of the glymphatic system (Hubbard et al., 2015). The expression of perivascular AQP4 decreased over time and gradually recovered with the degree of injury in 14–28 days in the mouse ischemic injury model. The pattern of AQP4 localization changes was consistent with the occurrence and regression time of cerebral edema (Wang et al., 2017). Concurrently, the solute clearance in the infarct core of mice was still impaired after recovery from the injury (Wang et al., 2017), and the injury was related to the prognosis of the mice, which suggested that the glymphatic system might be related to the recovery of edema after stroke in various dimensions.

#### The Inflammatory Response in the Perivascular Space

After ischemic stroke, stimulation by hypoxia, cell debris, and cytokines can lead to the activation of microglia and astrocytes, as well as the production of inflammatory cytokines (Zhou et al., 2017). In addition, perivascular astrocytes can also cooperate with microglia to produce a majority of the complement and cytokines, which are secreted directly into the CSF through the perivascular space (Pagenstecher et al., 2000; Williams et al., 2020). Furthermore, the inflammatory response in the perivascular space, as a powerful mechanism to promote brain edema, can also exacerbate BBB destruction, further leading to brain swelling (Daneman and Prat, 2015). Therefore, in vasogenic edema, the inflammatory response in the perivascular space may be an important contributor to swelling of the brain parenchyma. The glymphatic system has a dual role in the inflammatory response of the CNS (Louveau et al., 2015a). On one hand, the glymphatic system exacerbates brain edema by promoting perivascular inflammation. CNS damage leads to the proliferation and activation of T cells in the deep neck lymph nodes (Walsh et al., 2015), which may be caused by the drainage of molecules and cells from the damaged site to the deep neck lymph nodes through the glymphatic system and meningeal lymphatic vessels. On the other hand, it also facilitates the recovery of the BBB and CNS function by promoting the removal of inflammatory factors and cell fragments in the late stage of edema (Willis et al., 2013).

### Pathophysiological and Molecular Mechanisms of the Glymphatic System in Hemorrhagic Stroke

#### The Changes of Perivascular Space After Hemorrhagic Stroke

Fibrin in the paravascular space and impaired inflow of the glymphatic system are important mechanisms for the formation of brain edema in hemorrhagic stroke. Unlike an ischemic stroke, the BBB in hemorrhagic stroke is open at the beginning, and a large number of blood cells and coagulation factors enter the perivascular space and brain parenchyma with hydrostatic pressure and osmotic gradient. The internal flow of the glymphatic system is severely impaired in the acute phase of subarachnoid hemorrhage (SAH). Moreover, using MRI to evaluate the SAH model of mice, the influx of the ipsilateral glymphatic system decreased significantly at 24 h, and this decrease in influx flow of the glymphatic pathway persists after bilateral craniectomy (Gaberel et al., 2014), which suggests that it is not associated with intracranial hypertension induced by SAH. High-molecular-weight fluorescent dyes (FITC-dextran) were found to accumulate in the perivascular space after injection into the brain, but they could not pass through (Gaberel et al., 2014). In another study, the researchers injected a fluorescent tracer into the mouse cistern, and immunohistochemical samples confirmed the presence of a large amount of fibrin/fibrinogen in the perivascular space after SAH (Golanov et al., 2018). This indicated that the fibrin/fibrinogen produced in the coagulation cascade blocks the perivascular space, leading to functional impairment of the glymphatic system. Fluorescent tracer drainage into the brain parenchyma and deep cervical lymph nodes was also decreased at 1 week after SAH surgery (Golanov et al., 2018). However, it is worth noting that the influx flow of CSF is not related to the amount of bleeding. Additionally, deposition of fibrin/fibrinogen and reduction of CSF influx were detected in non-bleeding areas (Golanov et al., 2018). Both tissue plasminogen activator and cerebral coagulation factor III antibody can reduce the deposition of fibrin/fibrinogen around blood vessels and restore the function of the glymphatic system (Golanov et al., 2018). The evidence implies that it is not the SAH itself, but the induced production of fibrin/fibrinogen deposition that leads to dysfunction of the glymphatic pathway.

#### The Changes of AQP4 After Hemorrhagic Stroke

In the mouse model of SAH, the flow of fluorescent tracer into the brain parenchyma and the deep neck lymph nodes is reduced, and the polarization state of AQP4 changes, which means that the accumulation of blood cells and blood components after SAH affects the normal function of the glymphatic system and lymphatic clearance pathway (Pu et al., 2019). After using deferoxamine to reduce the iron concentration around the hematoma, the expression of AQP4 decreases, and brain edema is reduced in the model of experimental intracerebral hemorrhage (ICH; Qing et al., 2009). AQP4 knockout mice in the SAH model, which was established using the endovascular perforation method, showed impaired transport of ISF and increased water content in the brain (Liu et al., 2020). The contradictions between the two studies may be related to the difference in SAH models, or it may mean that AQP4 has a biphasic effect after a hemorrhagic stroke. In addition, neither of the two studies involved AQP4 polarization and depolarization, suggesting that the polarization of AQP4 and its specific mechanism should be further studied in SAH.

## Molecular Targets of The Glymphatic System in The Treatment of Cerebral Edema After Stroke

The glymphatic system plays an important role in the recovery of ischemic brain edema. The conventional viewpoint is that the ions and water produced in brain edema leave the brain mainly through the space of the vascular endothelium (Damkier et al., 2013). The opening of the BBB after stroke is more conducive to the flow of water and ions into the brain parenchyma along with the hydrostatic pressure and osmotic gradient (Ishimaru et al., 1993). Moreover, during the recovery period of brain edema, BBB closure often takes precedence over the regression of edema (MacAulay and Zeuthen, 2010). Several studies have shown that the glymphatic system can remove excess water, ions, and a variety of solute molecules from brain tissue (Iliff et al., 2012, 2013b; Kress et al., 2014). On one hand, the degree of brain edema is reduced by the drainage of water, ions, and proteins from glymphatic pathways. On the other hand, with the alleviation of brain edema, the function of the glymphatic system gradually recovers, which in turn promotes the recovery of the CNS from brain edema and other pathological states. The recovery of the glymphatic system is related to the long–term prognosis of patients with brain edema after ischemic stroke. The glymphatic system in a mouse model of multiple microinfarctions, induced by intraarterial injection of cholesterol crystals, recovered after 14 days, but the core of the infarct area did not recover and the solute remained (Wang et al., 2017). This may explain the long–term degenerative neuropathy that occurs in patients with ischemic stroke. In conclusion, water, ions, and metabolic waste from brain edema after ischemic stroke, at least in part, exit the brain tissue through the glymphatic pathway, facilitating the recovery of brain edema (Cerase et al., 2010).

The discovery of the glymphatic system has increased understanding of brain transport pathways. Furthermore, the glymphatic system is not isolated, as it plays a role in the formation and regression of brain edema by interacting with other transport pathways of solutes and liquids in the CNS (Stokum et al., 2016). Broadly speaking, the study of brain edema is the study of the transport pathway of solutes and liquids in the CNS. Various molecules, including AQPs, transporters, ion channels, and vascular permeability factors, are thought to be associated with cytotoxic and vasogenic edema after CNS damage (Michinaga and Koyama, 2015). In recent studies, these molecules have become the focus of targeting edema drugs, some of which are described in this section (Table 1).

**Table 1:** Treatments of post-stroke edema by regulating the glymphatic system.

Mechanism	Drug		Effect	Reference
Water channel protein inhibitors	TEA		Block the water permeability of AQP1, AQP2, and AQP4	Detmers et al. (2006)
	carbonic anhydrase inhibitors	AZA	Reduce AQP4 water permeability	Huber et al. (2007)
		EZA		
		4-acetamidobenzsulfonamide		
		TGN-020	Reduce cerebral edema in mice and decrease cerebral infarction	Huber et al. (2009)
		MB	Improve brain edema and astrocyte swelling	Shi et al. (2021)
Transporters and ion channel inhibitors	NKCC1inhibitors	BMT	Relieve the volume and improve the prognosis of brain edema in hemorrhagic or ischemic animal models	Yan et al. (2003), Wang G. et al. (2014), and Xu et al. (2017)
		STS66	Alleviate the volume and functional recovery of brain edema after transient ischemic stroke	Huang et al. (2019)
	Sur1-Trpm4 channel inhibitor	Glibenclamide	Reduce the swelling of the cerebral hemisphere and restore neurologic function after stroke	Simard et al. (2006), Simard et al. (2009), Ortega et al. (2012), Wali et al. (2012), Ortega et al. (2013), Vorasayan et al. (2019), Zhao and Huang (2019), and da Costa et al. (2019)
		PACAP	Down-regulate AQP4 by inhibiting the expression of Sur1and attenuate cerebral edema after SAH	Fang et al. (2020)
Inhibition of vascular permeability factor expression	MMP9	BB-1101	Reduce brain edema and permeability of BBB	Rosenberg and Navratil (1997), Rosenberg et al. (1998), and Sood et al. (2008)
	VEGF	Bevacizumab	Reduce brain edema and improve symptoms caused by radiation-induced brain necrosis in patients undergoing brain tumor resection	Fraum et al. (2011)
		VGA1155	Reduce the volume of brain edema in rats with frozen injury	Koyama et al. (2007)

*AZA, acetazolamide; EZA, ethoxazolamide; TGN-20, intraperitoneal pre-injection of 2-nicotinamide-1,3,4-thiadiazole; MB, methylene blue, NKCC1, Na^+^-K^+^-Cl^−^co-transporter1; BMT, bumetanide; STS66:3-(Butylamino)-2-phenoxy-5[(2,2,2-trifluoroethylamino) methyl] benzenesulfonamide; PACAP, pituitary adenylate cyclase-activating polypeptide*.

### Aquaporin Family

AQP1, AQP4, and AQP9 are the main aquaporins comprising the AQP family in the CNS (MacAulay and Zeuthen, 2010). AQP4, as the most widely studied aquaporin in the CNS and an important component of the glymphatic system, plays an important role in promoting the flow of solutes and liquids in CSF and ISF (Mestre et al., 2018a). AQP4 is quite complex in the pathogenesis of brain edema. Deletion of the AQP4 gene alleviates cytotoxic edema in ischemic stroke mice (Manley et al., 2000; Haj-Yasein et al., 2011). However, in the ICH model, AQP4-deletion mice showed more severe brain edema than wild-type mice (Papadopoulos and Verkman, 2013). The above results suggest that deletion of AQP4 may increase the resistance of CSF-ISF exchange through the glymphatic pathway, and may reduce the flow of CSF entering the brain parenchyma and brain cells during cytotoxic edema, but may also slow down the clearance rate of excess fluid in vasogenic edema (Papadopoulos and Verkman, 2007, 2013).

A variety of AQP inhibitors have been found and their possible effects against brain edema effects have been verified in some experiments. Tetraethylammonium (Tea) is proven to be an inhibitor of AQP1, AQP2, and AQP4 in oocytes *in vitro* (Detmers et al., 2006). While both AQP1 and AQP4 play an important role in brain edema, nonspecific inhibitors may impact the effect of attenuating brain edema. Many carbonic anhydrase inhibitors, including acetoacetate, are also thought to exert AQP4 inhibitory effects (Huber et al., 2007). The functional *in vitro* experiments confirmed that carbonic anhydrase inhibitors, such as acetazolamide (AZA), ethoxazolamide (EZA), and 4-acetamidobenzsulfonamide, reduced the water permeability of the AQP4 channel by 80%, 63%, and 23%, respectively (Huber et al., 2007). However, this study did not conduct further animal experiments to verify the exact effect of these inhibitors in brain edema models. MRI results showed that intraperitoneal pre-injection of 2-nicotinamide-1,3,4-thiadiazole (TGN-20), a novel AQP4 inhibitor, can effectively reduce cerebral edema and the range of cerebral infarction in mice (Huber et al., 2009). Similarly, methylene blue (MB) can improve brain edema and astrocyte swelling by inhibiting AQP4 expression, which is a potential drug choice in AQP4-targeted treatment (Shi et al., 2021). Generally speaking, although potential anti-edema drugs targeting AQP4 have been found, there is limited evidence in *in vitro* and *in vivo* experiments, and no evidence of systematic and prospective clinical trials of these drugs against brain edema.

### Ion Transporters and Ion Channels

There are extensive ion transporters and ion channels in astrocytes and endothelial cells of the CNS. Under physiological conditions, these ion transporters and ion channels are driven by active transport. Secondary transport of various ions and water, together with active transport, maintains the homeostasis of the CNS.

Na^+^-K^+^-Cl^−^ co-transporter1 (NKCC1) is constitutively expressed in astrocytes in various regions of the adult brain, mediating electrically neutral transport of chloride ions, potassium, and/or sodium on the membrane (Kahle et al., 2009; Jayakumar and Norenberg, 2010; Rabinstein, 2010). NKCC1 transport activity was upregulated in mouse models of cerebral infarction, possibly due to phosphorylation and up-regulation of protein expression (Yan et al., 2003; Simard et al., 2010). Only one study found that bumetanide (BMT) did not alter the volume of edema and neurological function in rats with ICH (Wilkinson et al., 2019), and a number of experiments have confirmed that BMT can relieve the volume and improve the prognosis of brain edema in hemorrhagic or ischemic stroke animal models by inhibiting NKCC1 (Yan et al., 2003; Wang G. et al., 2014; Xu et al., 2017). In a recent study, a novel NKCC1 inhibitor, STS66 (3-(Butylamino)-2-phenoxy-5[(2,2,2-trifluoroethylamino)methyl]benzenesulfonamide), has been shown to play a positive role in alleviating the volume and functional recovery of brain edema after transient ischemic stroke (Huang et al., 2019).

The Sur1-Trpm4 channel is a non-selective cationic channel that is activated only after injury in the CNS, and almost no expression exists within a healthy brain (Simard et al., 2006, 2012). Some studies have shown that the sulfonylurea drug, glibenclamide, can alleviate brain edema by inhibiting the Sur1-Trpm4 pathway. In many experiments, glibenclamide can significantly reduce the swelling of the cerebral hemisphere and restore neurological function after stroke (Simard et al., 2006, 2009; Ortega et al., 2012, 2013; Wali et al., 2012). In many clinical trials, glibenclamide can reduce infarct size and improve neurological function after ischemic stroke (Vorasayan et al., 2019; Zhao and Huang, 2019). Clinical trials for the efficacy of glibenclamide in patients with aneurysmal SAH are also ongoing (da Costa et al., 2019). In conclusion, ion transporters and ion channels are potential targets for brain edema, and specific drugs, such as budesonide and glibenclamide, are expected to provide new methods for the treatment of brain edema. In a study of a brain edema model of cerebellar injury, Sur1-Trpm4 and AQP4 form a complex to aggravate astrocyte swelling (Stokum et al., 2018). A recent study shows that pituitary adenylate cyclase-activating polypeptide (PACAP) can downregulate AQP4 by inhibiting the expression of Sur1, thereby attenuating brain edema after SAH (Fang et al., 2020).

### Vascular Permeability Factor

Many cytokines, including MMP9 and VEGF, can lead to TJ protein destruction and increased BBB permeability, which play an important role in vasogenic brain edema after CNS injury (Seo et al., 2012; Geiseler and Morland, 2018). Expression of MMP9 was observed in astrocytes, microglia, neurons, and endothelial cells in the CNS (Lee et al., 2004; Jia et al., 2010). Wide-spectrum MMP inhibitors (BB-1101) have been shown to reduce brain edema and permeability of BBB in animal models, such as cerebral ischemia and cerebral hemorrhage (Rosenberg and Navratil, 1997; Rosenberg et al., 1998; Sood et al., 2008), although BB-1101 may be a cause of a significant neurological deterioration in treated animals (Sood et al., 2008).

VEGF is commonly expressed in astrocytes, neurons, and endothelial cells (Hayashi et al., 1997; Mărgăritescu et al., 2011). and can play a neuroprotective role in ischemic stroke (Shim and Madsen, 2018). However, VEGF can also increase BBB permeability by destroying the TJ protein of the BBB (Argaw et al., 2009). During cerebral ischemia or trauma, immunohistochemistry showed that expression of the VEGF protein was up-regulated (Sköld et al., 2005; Mărgăritescu et al., 2011). The VEGF monoclonal antibody, bevacizumab, has been shown to reduce brain edema and improve symptoms caused by radiation-induced brain necrosis in patients undergoing brain tumor resection (Fraum et al., 2011). However, the efficacy of bevacizumab against brain edema in ischemic and hemorrhagic stroke requires further clinical verification. Moreover, VEGF antagonists, such as VGA1155, could reduce the volume of brain edema in rats with frozen injury (Koyama et al., 2007).

Other than MMP9 and VEGF, the expression of many proteins, such as chemokine CCL2, angiogenin 2 (Ang2), and nitric oxide (NO), was upregulated after CNS injury and was involved in inhibiting the expression of TJ proteins, thus exacerbating the formation of vasogenic edema (Keep et al., 2018). These molecules may be an important target to prevent vasogenic edema after injury, which is expected to provide a new method for treating brain edema.

## Conclusion and Perspectives

The glymphatic system plays an important role in the formation and recovery of edema after stroke, and is also an indispensable element for the expansion of post-stroke inflammation, as well as the removal of inflammatory cytokines and cell debris. The glymphatic system can affect the prognosis of stroke through these complex pathophysiological processes. At present, drug studies on aquaporins, ion channels, and vascular permeability factors have confirmed that regulating the glymphatic system can promote recovery and neurological function of brain edema after stroke, respectively. Further exploration of the pathophysiological and regulatory mechanisms of the glymphatic system in brain edema after stroke will be helpful in providing an innovative target and direction for treatment. More clinical trials are needed to validate the drugs targeting the regulation of the glymphatic system. Moreover, the effects and long–term prognoses are deserving of further research.

## Author Contributions

XZ and YH conceived the main outline. XZ wrote the manuscript. XZ and YL made the figures. CL, MW, and YO took charge for manuscript revision in English. YH participated in the correction and final review of this article. All authors contributed to the article and approved the submitted version.

## Conflict of Interest

The authors declare that the research was conducted in the absence of any commercial or financial relationships that could be construed as a potential conflict of interest.

## Publisher’s Note

All claims expressed in this article are solely those of the authors and do not necessarily represent those of their affiliated organizations, or those of the publisher, the editors and the reviewers. Any product that may be evaluated in this article, or claim that may be made by its manufacturer, is not guaranteed or endorsed by the publisher.
